# The role of DNA mismatch repair in immunotherapy of human cancer

**DOI:** 10.7150/ijbs.71714

**Published:** 2022-04-04

**Authors:** Yuchen He, Luyuan Zhang, Ruoyu Zhou, Yumin Wang, Hao Chen

**Affiliations:** 1 Department of General Surgery, The First Affiliated Hospital of USTC; Division of Life Sciences and Medicine, University of Science and Technology of China, Hefei, Anhui, China.; 2 Xiangya School of Medicine, Central South University, Changsha, Hunan, China.; 3 Department of Neurosurgery, First Affiliated Hospital, School of Medicine, Zhejiang University, Hangzhou, Zhejiang, China.; 4 Department of Otolaryngology Head and Neck Surgery, Xiangya Hospital, Central South University, Changsha, Hunan, China.; 5 National Clinical Research Center for Geriatric Disorders, Xiangya Hospital, Central South University, Changsha, Hunan, China.

**Keywords:** MMR, Development, Immunotherapy, DNA repair, Cancer

## Abstract

DNA mismatch repair (MMR) is an important pathway which helps to maintain genomic stability. Mutations in DNA MMR genes are found to promote cancer initiation and foster tumor progression. Deficiency or inactivation of MMR results in microsatellite instability (MSI) which triggers neoantigen generation and impairs tumor growth. Immunotherapies targeting MMR can increase the burden of neoantigens in tumor cells. While MSI has been regarded as an important predictor of sensitivity and drug resistance for immunotherapy-based strategies. Different approaches targeting genomic instability have been demonstrated to be promising in malignancies derived from different tissues. Underlying MMR deficiency-associated immunogenicity is important for improving the therapeutic efficacy of immunotherapies. In this review we provide an overview of the MMR systems, their role in tumorigenesis, drug resistance, prognostic significance and potential targets for therapeutic treatment in human cancers, especially in hematological malignancies.

## Background

Cancer ranks first in the cause of morbidity in the world. The global cancer burden is expected to be 28.4 million cases in 2040, a 47% rise from 2020, with a larger increase in transitioning (64% to 95%) versus transitioned (32% to 56%) countries due to demographic changes 1. The rising prominence of cancer makes it a leading cause of death which partly reflects marked declines in mortality rates of stroke and coronary heart disease 2. Overall incidence was from 2‐fold to 3‐fold higher in transitioned versus transitioning countries for both sexes 3, 4. Cancer incidence and mortality are rapidly growing worldwide, with a predicted 22 million new cancer cases and 13 million cancer-related deaths occurring annually by 2030 5.

The occurrence and development of cancer is a complex process of multi-stage, multi-factor, long-term exposure and multi-channel accumulation, caused by genetic and environmental factors 6,7. The hallmarks of cancer have provided a framework for a deeper molecular understanding of cancer which include sustaining proliferative signaling, evading growth suppressors, resisting cell death, enabling replicative immortality, inducing angiogenesis, activating invasion and metastasis, reprogramming of energy metabolism, tumor-promoting inflammation, avoiding immune destruction 8. Genome instability, which generates the genetic diversity that expedites their acquisition, participates in the regulation of these hallmarks and inflammation.

As its name indicates, DNA mismatch repair (MMR) is responsible for correcting the mismatched nucleotides caused by polymerase misincorporation errors, recombination between heteroallelic parental DNAs, and chemical or physical damages 9. To achieve the repairment, MMR goes through an excision-resynthesis process that requires the cooperation of MMR protein complex, DNA replicative polymerase and DNA ligase 10. In addition to its roles in editing replication errors, the MMR system is also implicated in the DNA damage response (DDR) that triggers cell cycle arrest and apoptosis, avoiding tumorigenesis caused by unrepairable damages 11. Mutations of human MMR genes were linked to common human cancers 12-14. According to the data from The Cancer Genome Atlas (TCGA), most mutations found in human tumors are single base substitutions 15. This high mutational burden renders tumors immunogenic and sensitive to programmed cell death-1 (PD-1) immune checkpoint inhibitors 16. Besides, patients with MMR-deficient tumors experience highly variable responses, and roughly half are refractory to treatment 17. These discoveries solidify a role for MMR in human tumorigenesis and provide support for the hypothesis that mutators might be driving the large numbers of mutations found in cancer. This article intends to review the function and mutation of MMR genes and their roles in cancer immunotherapy.

## The mechanism of MMR gene causing tumor

**Figure 1:**
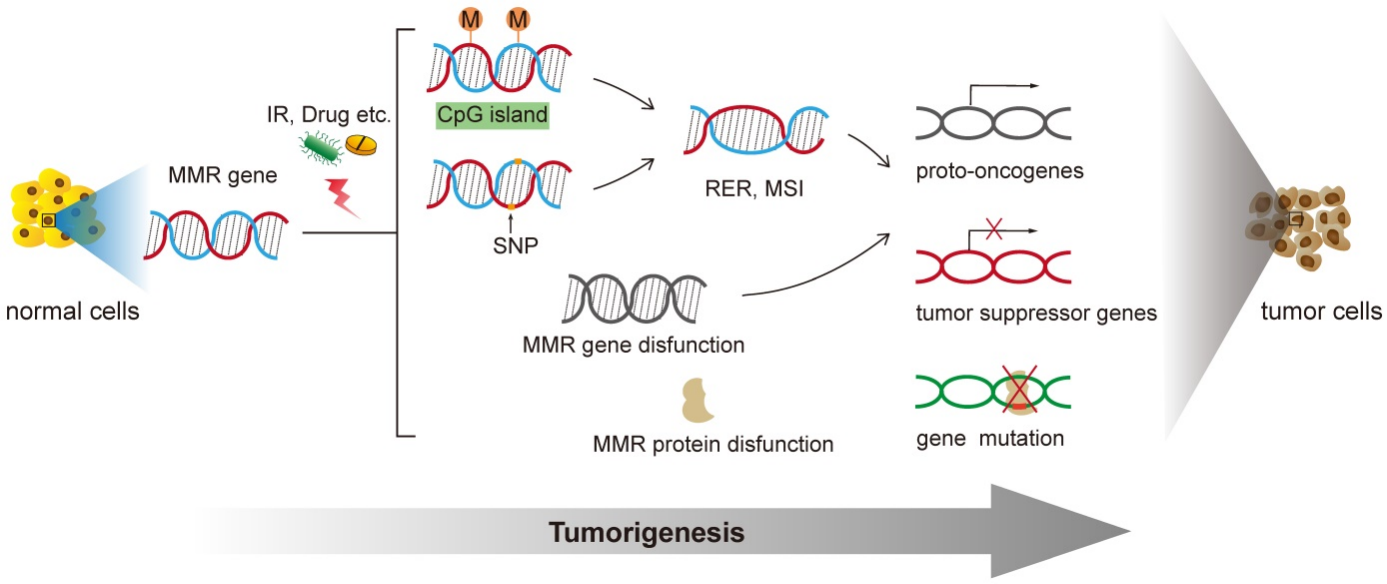
Functions of DNA Mismatch repair in tumorigenesis of human cancer.

The MMR gene is a group of genetic susceptibility genes isolated from hereditary non-polyposis colorectal cancer (HNPPC) 18. Fisher isolated and cloned the first human MMR gene hMSH2 in 1993. At present, totally nine genes were found involved in the function of mismatch repair: mutS homologs (MSH2, MSH3, MSH4, MSH5, MSH6), mutL homologues (MLH1, MLH3), and postmeiotic segregation increased (PMS1, PMS2) 19. In the process of biological evolution, MMR genes remain conservative, mainly functioning to correct the base mismatch that are generated during DNA recombination and replication. Properly functioned MMR genes ensure the integrity and stability of genetic material, induce apoptosis of DNA damaged cells and eliminate the formation of mutant cancerous cells 20. Any mutations of this gene family will lead to defects in the mismatch repair function, cause genetic instability, and eventually lead to tumorigenesis 21 **(Figure 1).**

### MMR gene promoter methylation

Some carcinogens, such as microorganisms and alkylating agents, can inhibit the MMR gene transcription process by methylating the CpG island in the promoter region and preventing the action of transcription factors 22. Analysis results from 32 patients showed that the methylation rate of hM-SH2 promoter region in esophageal cancer tissue was 34.4%, and no methylation was found in normal esophageal tissue 23. In liver cancer specimens, the methylation rate of CpG island in hMSH2 promoter is up to 68.4%, making it a common early genetic change in the occurrence and development of liver cancer 24. The methylation rate of the hMLH1 promoter CpG island was 72.9% in gastric cancer tissues compared with 20% in non-gastric cancer tissues 25. This significant difference indicates that methylation is highly related to the occurrence and development of gastric cancer 26. Methylation of hMLH1 gene could be detected in 89% of patients with endometrial cancer 27, 18.0% in elderly colon cancer samples by Polymerase chain reaction (PCR) and 51.2% through the heterozygous deletion of microsatellite markers 28. These studies have shown that the promoter methylation of DNA mismatch repair genes exists in most tumors, especially hMSH2 and hMLH1.

### MMR gene polymorphism and microsatellite instability

A single nucleotide polymorphism (SNP) refers to an orthologous nucleotide position that is variable across the genomes. Generally they are base-pair differences among chromosome sequences which are caused by mutations that convert, transverse, insert or delete single bases 29. More than 90% essential manifestation of human genetic information is caused by genetic polymorphism 30. SNPs in MMR genes are permanent changes that can impair mismatch repair function. When this happens, MMR system cannot repair mismatched bases and insertion/deletion loops. As a results, a large number of replication errors (RER) and MSI are generated during DNA replication 31. The non-repetitive single-base DNA sequences are mainly affected by base-base mismatches. In addition, the insertion or deletion of short repeat sequences such as insertion-deletion loops is another reason for the occurrence of MSI 32.

MSI is a common phenomenon observed across different solid tumor types. Examples of common cancers that have MSI-H frequency >10% include colorectal cancer, endometrial cancer, and gastric cancer. Cancers with MSI-H frequency between 2% and 10% include ovarian cancer, cervical cancer, and thyroid cancer 21. The polymorphisms of hMLH1 at -93G-A were detected in 165 patients with non-small cell lung cancer and 193 healthy controls 33. Results showed that the homozygous variant A/A genotype was associated with a significantly increased risk for lung cancer. The patients with a homozygous variant A/A genotype had a trend toward poorer prognoses compared with other patients 33. PCR-SSCP was applied to detect the MSI gene status of small intestinal adenocarcinoma tissues and adjacent tissues. Among them, MSI occurred in 19 cases (32.76%) of cancer tissues, and 9 cases had hMLH1 and hMSH2 gene mutations. The total rate was 47.37%, but no mutations were found in those who did not have MSI. Therefore, hMLH1 and hMSH2 gene mutations have clinical significance for the early diagnosis of small intestinal adenocarcinoma.

### Change the mutation frequency of oncogenes and/or tumor suppressor genes

Besides oncogenes and tumor suppressor genes, MMR genes are the third type of genes that are closely related to tumorigenesis. Studies have shown that MMR dysfunction leads to the loss of repairment of the mutated coding region, directly or indirectly leading to the activation of proto-oncogenes and the inactivation of tumor suppressor genes 9. As a result, cells begin to proliferate and differentiate indefinitely, eventually causing tumorigenesis. It's reported that people who positively express hMSH2 genes have a lower expression level of p53 compared with those who negatively express hMSH2 genes 34. Speculating that the hMSH2 gene could be used as a protective factor, it might function via p53-dependent pathways.

### Defects in MMR protein function

Of the 9 human MMR genes, six are involved in the mismatch repair process, namely hMSH2, hMSH3, hMSH6, hMLHl, hPMSl and hPMS2. MSH2 couples with either MSH6 or MSH3 (forming MutSα and MutSβ complexes, respectively), and MLH1 couples with PMS2 or MLH3 (forming MutLα, MutLβ or MutLγ complexes, respectively). hMutL forms a temporary complex with hMutS and bound to the DNA strand, responsible for the recognition of mismatches and insertion-deletion loops 35, 36. Then recruitment of the MLH1/PMS2 complex will degrade the mutated stretch and initiates resynthesis. The newly formed DNA strand replaces the excised mismatched DNA strand to complete the repair process.

The reduction or deletion of MMR leads to defects in DNA repair, which affects the normal progress of DNA replication and increases the risk of developing tumors. Mismatch repair gene SNPs were found in most individuals with MMR gene expression defects 37. In addition, the expression of hMSH2 was positively correlated with the malignant level of liver cancer 38. MMR gene mutations may also form truncated proteins, stopping the transcription and translation.

### Other mechanisms

Related mechanisms also include activity changes of cytokines caused by the mismatch repair gene SNPs such as IFN-beta 39. These cytokines control the regulation of cell cycle and inflammation. Once lost, malignant proliferation of tumor cells would get started. However, the detailed mechanism still needs to be researched in future.

## MMR gene polymorphism and cancer

### The structure of hMSH2 and its relationship with cancer

The hMSH2 gene is in the chromosomal region 2p21, which covers a 73Kb segment with 16 exons. hMSH2 gene has molecular weight corresponding to 104,7 KDa, encoding a nuclear localization protein containing 934aa 40. MMR is a powerful, evolutionary conserved, mutation avoidance mechanism. The identity between hMSH2 and the corresponding region of the yeast protein is 85%. hMSH2 protein can specifically bind the mismatch of G-T and A-C in the DNA double-strand and can bind to (CA) n and 14-base insertion-deletion protruding loops. In humans, DNA mismatches are recognized by one of two heterodimers, both of which contain MSH2. MutSα (MSH2-MSH6) preferentially recognizes and repairs base-base mismatches as well as small insertion and deletion loops, whereas MutSβ (MSH2-MSH3) recognizes and repairs small insertion and deletion loops 41. The intron mutation rate of hMSH2 gene is higher than that of exon, so the existing reports are mostly concentrated in the intron region.

Mutations in the MSH2 gene are linked with the Lynch syndrome (LS), also known as HNPPC, hematological malignancy, gastrointestinal, urinary tract and ovary cancers 42-44. Besides, MSH2 mutations are found in patients with endometrial carcinoma and adenocarcinoma of the colon. Besides, MSH2 mutation carriers have an increased risk of breast cancer (BC) with or without a LS family history 45. A homozygous G to A transition mutation in the invariant G of the intron 10 splice acceptor of the MSH2 gene is associated with leukemia and multiple cafe´-au-lait spots, a feature of neurofibromatosis type 1 46. Three sites of hMSH2 gene: rs2303428, rs4952887 and rs2059520 in liver cancer samples. Results showed that in the development of liver cancer, polymorphism of rs2303428 site of hMSH2 gene was related to HBsAg positive and hepatic tumor family history. There is no interaction between rs4952887 and rs2059520.

Mechanically, researchers have found that MSH2 mutations altered the regulating pathways of nuclear factor erythroid 2-related factor 2 (Nrf2). Nrf2 is an important regulator in modulating DNA mismatch repair (MMR) gene in acute myeloid leukemia (AML). Studies have found that patients with Nrf2 overexpression had a higher frequency of gene mutation and drug resistance. Mechanism behind is that Nrf2 overexpression inhibited MSH2 protein expression in a ROS-independent manner via JNK/c-Jun signaling, which caused DNA MMR deficiency and induced gene instability-dependent drug resistance in AML 47.

### The structure of hMLH1 and its relationship with cancers

The hMLH1 gene is a mismatch repair gene discovered after hMSH2 in 1994 48. It is located on chromosome 3p21 and is homologous to the MutL in bacteria. Its genome is 58 kb in length and contains 19 exons. The total length of cDNA of hMLH1 is 2484bp. The 2268bp development reading frame of hMLH1 encodes protein containing 756 amino acids, and 41% homology with hMLH1 of yeast 49. hMLH1 is a potential functional SNP. The expression of hMLH1 protein is affected by SNP and is closely related to the occurrence and development of tumors. At present, research on hMLH1 and human cancers are relatively extensive.

**Figure 2:**
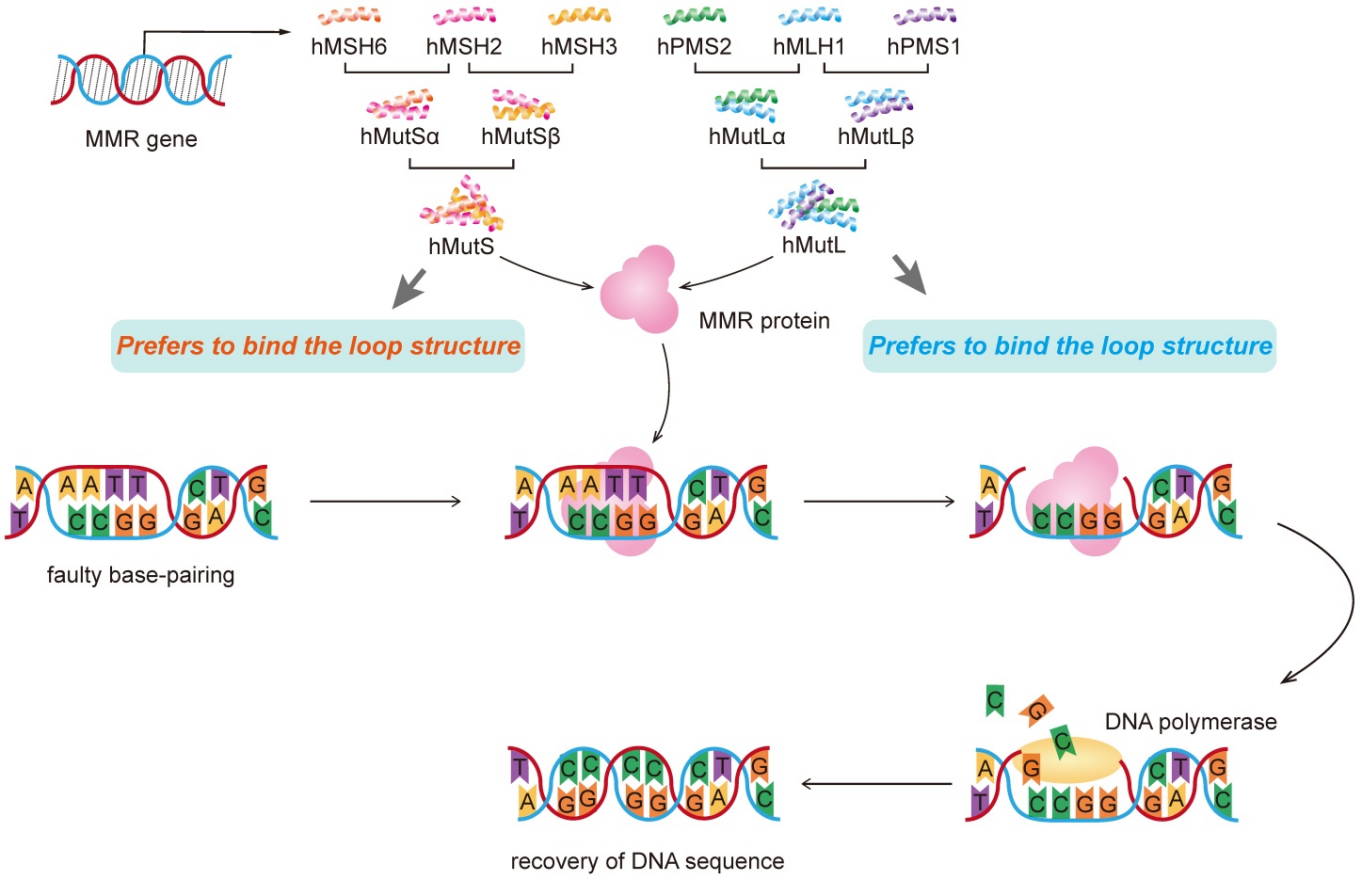
Molecular mechanism of DNA mismatch repair with represent MMR genes.

A homozygous germ-line MLH1 mutation was found to cause a mutator phenotype characterized by leukemia and/or lymphoma associated with neurofibromatosis type 1 50. In addition to mutations, hypermethylation of the MLH1 gene promoter and subsequent mismatch repair deficiency were involved in the pathogenesis of hematological malignancies such as acute T-cell leukemia/lymphoma (ATL) 51, 52. Moreover, a study of 453 cases showed that after adjusted for age and gender, individuals with AG and GG genotypes in hMLH1 gene rs1800734 had higher risk than that of AA genotype in developing liver cancer. The frequency of allele A in the case group was higher than that in the control group. There are different major binding features for MMR genes. MutS alpha complex prefers to bind mismatch while MutSbeta prefers to bind the loop structure** (Figure 2).**

In addition, there may be a gene-environment interaction between the hMLH1 gene polymorphism and HBV infection and family history of tumors but not with drinking. In the occurrence and development of liver cancer, the polymorphism of hMLH1 locus rs1800734 gene is related with tumor family history and HBsAg positive 53. The expressions of hMSH2 and hMLH1 are positively correlated with the degree of differentiation of liver cancer tissues. The higher the degree of differentiation, the higher the expression level. Reduced expression of hMLH1 gene was found in high-grade hepatocellular carcinoma 54.

### The structure of hMSH6 and its relationship with cancer

In 1995, it was discovered that one of the members of the MMR family is hMSH6, which combined with hMSH2 protein to constitute the mismatch repair hMutSα protein complex 55. hMSH6 protein is responsible for G/T mismatch, naming as G/T mismatch binding protein (GTBP). hMSH6 gene is 23806 bp in length and located on chromosome 2 p15-16, containing 10 exons and an untranslated region of 83 bp. The full-length cDNA is 4.2 kb, and the protein is 160kD. Studies had found that it mainly affected the stability of the human genome, and most of the mutations appear as small base mutations 56.

MSH6 mutations are related to several diseases. For example, MMR-deficiency (MMR-D) syndrome is characterized by childhood brain tumors, hematological and/or gastrointestinal malignancies, and signs of neurofibromatosis type 1 (NF1). Using an RNA-based mutation detection assay, researchers found a homozygous complex MSH6 splicing alteration in the index patients of a family with children suspected to MMR-D syndrome 57. A germline de novo 2p16.3 deletion of MSH6 was found in a boy with neurodevelopmental delay and a diffuse large B-cell lymphoma (DLBCL) 58. A case-control study involving 250 patients showed that the CT genotype of hMSH6 (rs1042821) reduced the risk of liver cancer. Most studies on hMSH6 gene are accompanied with hMSH2 gene, mainly because hMSH2 protein and hMSH6 protein combine to form heterodimer hMutSα protein to achieve the mismatch repair function. The polymorphism of hMSH6 gene alone is not enough to cause an increase in cancer susceptibility.

In addition to influencing tumorigenesis, MSH6 mutations are also involved in drug resistance. Relapse-specific heterozygous deletions in MSH6 results in a hypermutator phenotype associated with generation of secondary mutations that contributes to the development of thiopurine resistance in pediatric B-lymphoblastic leukemia 59.

### The function of other MMR genes and the relationship with cancer

Other five genes, hMSH3, hMSH4, hMSH5, hPMS2 and hMLH3, achieve their function via working with other proteins: hMSH3 and hMSH2 form a complex hMmtSβ, which mainly recognizes 2-4 base mismatches; hMSH4 and hMSH5 mainly participate in meiotic recombination 60; hPMS1, hPMS2 and hMLH1 form complexes hMutLβ and hMutLα, respectively; hMLH3 and hMLH1 form complexes 61.

## The function of MMR in the immunotherapy of human cancer

### Development of immunotherapy in cancer

Historically, conventional chemotherapies and radiotherapies have been identified to inhibit the proliferation or cause the death of cancer cells based on their uncontrolled relentless proliferation capacity. However, these therapies are cytotoxic with non-specific targets, such as DNA itself or enzymes required for DNA synthesis and repair. These targets are not restricted to malignant cells but rather are common to most cell types, limiting the application 62. Because of this, people turn to human immune system, hoping to find some tumor-specific antigens as therapeutic targets. The concept that the immune system can specifically recognize and control tumor growth can be traced back to 1893 when William Coley used live bacteria as an immune stimulant to treat cancer 63. Tumor-specific antigens that are generated by somatic mutation can influence immune system response to carcinoma cells and contribute to tumor shrinkage. In the past decade there has been an explosion of new approaches and technologies to explore the human immune system with unprecedented precision. Tremendous progress has been made in the understanding of how immune system recognize cancer cells and how cancer evades the immune surveillance. These findings in turn offers new ways to stop cancer immune evasion and facilitate cleaning cancer cells 64.

Mammals have two major sub-immune systems. The innate immune system provides an immediate, but non-specific response, targeting broad groups of situations and stimuli. While the adaptive immune system provides an antigen-specific response and requires the recognition of specific "non-self" antigens during a process called antigen presentation 65. CD8+ cytotoxic T cells are important immune cells of adaptive immune system. They can recognize and target cancer cells that present tumor-specific antigens and drive the effect of tumor shrinkage. These antigens include but not limited to cancer testis antigens and somatic neoantigens 66. To activate tumor-specific immune responses, two fundamental requirements must be simultaneously fulfilled. First, cancer cells must express antigens that can be recognized by a circulating naive T cell clone. Second, malignant cells must deliver adjuvant-like danger signals to antigen-presenting cells (APC) in the form of exogenous microbe-associated molecular patterns (MAMPs) or as endogenous damage-associated molecular patterns (DAMPs) 67. The immune system encompasses inhibitory mechanisms to prevent excessive reactions and limit immune responses. These inhibitory mechanisms are necessary for balancing immunity in normal homeostasis. PD-1 is a member of the CD28/CTLA-4 family. This is a family of co-stimulatory receptors that expressed on the surface of natural killer cells, dendritic cells (DCs), activated monocytes, B cells and T cells. PD-1 contributes to the immune tolerance of self-antigens by conveying an inhibitory signal to T cells and suppressing immune response 68. However, in the presence of a growing malignancy, the balance is disrupted and skewed towards excessive inhibition of immune reactivity due to tumor-induced immune suppression and enhanced immunologic tolerance 69. Immunotherapies against inhibiting receptors, such as PD-1/PD-L1 antibodies, can block the combination of tumor PD-L1 and T cell PD-1, eliminating this immunosuppressive effect 70.

### Current immunotherapies in cancer

Immune checkpoint inhibitors are rapidly developing immunotherapy methods in recent years, which boost T-cell activity against patient-specific neoantigens, restoring the suppressed immune function of the body and killing tumor cells 71. During immune process, CD4+ regulatory T cells (Treg) accumulate, accompanied with CD8+ T cell activation. They regulate the duration and intensity of immune reaction by suppressing the function of effector CD8+ T cells 72. This mechanism is known as immune checkpoint, which helps to avoid excessive immune response. Antibody-based checkpoint blockade immunotherapy mainly acts by boosting the immune system to target tumor cells through releasing CD8+ T cells from immunosuppressive activity of Treg. Monoclonal antibodies targeting co-inhibitory immune checkpoints (e.g., PD-1 and CTLA-4) have demonstrated clinical activity in several malignances, including melanoma, non-small cell lung cancer, renal cell carcinoma, bladder cancer, head and neck squamous cell carcinoma, MSI-high colorectal carcinoma, Merkel cell carcinoma, and Hodgkin lymphoma 73. Approved treatments now include anti-PD-1 (nivolumab and pembrolizumab), anti-CTLA-4 (ipilimumab), and combination anti-PD-1/CTLA-4 regimens (nivolumab-ipilimumab) 74.

Cancer vaccines are another method to regulate immune system activity. Different from checkpoint inhibitors, cancer vaccines boost the immune system's ability to recognize and kill cancer cells by injecting cancer-specific elements into patients to 75. After injection, DCs take up and process the introduced antigens and display the antigen on the cell surface through major histocompatibility complex (MHC) class I or II molecules. Then DCs present these antigens to resting T cells and active them. Activated T cells start to proliferate and differentiate into CD8+ cytotoxic T cells and target antigens displayed on the tumor cell surface 76. One of the most widely adopted cancer vaccination is the design of MHC class I restricted peptide epitopes which are derived from shared tumor-associated antigens. Such vaccinations have been applied as experimental treatments for metastatic melanoma, clear cell renal cell cancer, melanoma/breast cancer and other tumor types. However, the attempt to use vaccines against chronic myelogenous leukemia (CML) by targeting the BCR-ABL fusion oncoprotein failed to prove a clear clinical benefit 77, 78. Reasons for the failure could be low antigen expression/presentation on these tumors that are not enough for T cells to initiate an appropriate immune response. Another reason could be that the tumor is rapidly able to adapt to immunologic selection via an immunoediting mechanism 79. Based on this, researchers started to use multiple, carefully selected shared neoantigens to see if it could increase the chance of inducing meaningful T-cell reactivity. Theoretically, this strategy could also lower the chances of tumor clones escaping from immune system elimination 80. Besides using tumor-associated peptides, other alternative antigen sources include antitumor dendritic cells, whole tumor cell mRNA extracts and tumor cell extracellular vesicles such as exosomes 81.

### MMR and cancer immunotherapy

**Figure 3:**
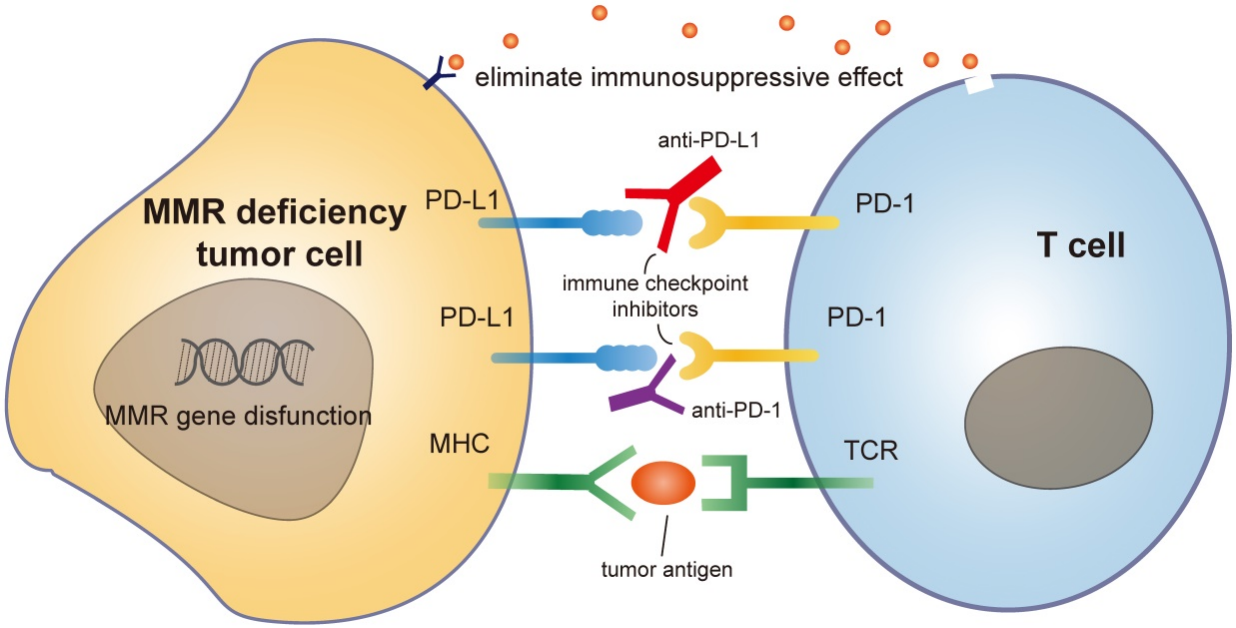
Mechanism of MMR deficiency tumor cell with immune checkpoint drug like PD1 antibody.

The mutation rate during replication is rather low, approximately once for every 104 and 105 nucleotides. Still, each time a cell divides, about 100,000 polymerase errors occur. Although DNA polymerases are able to correct part of these errors, some errors always escape proofreading, which need the help of MMR system 82. MMR gene mutation is common among different type of cancers. Due to these defects, cancer cells start to produce and secret mutated non-self-proteins which are termed as neoantigens. These neoantigens are recognized by the immune system and stimulate the activation of immune cells. These self-produced neoantigens can trigger a more robust and long-lasting immune response, suppressing tumorigenesis more effectively than externally injected neoantigen vaccines 83. Besides, MMR deficient tumors are immunogenic and sensitive to programmed cell death-1 (PD-1) immune checkpoint inhibitors 84, 85. An upregulation of genes involved in the immune response, such as proinflammatory cytokines and cytotoxic mediators is observed in MMR deficiency tumors, resulting in an increased secretion of soluble mediators in the tumor microenvironment with the subsequent activation of the PD-1 pathway 86. Studies on several types of cancers (colorectal, gastric, ovarian, upper urinary tract urothelial cancer, biliary tract cancers) have identified that MMR deficiency has a more favorable prognosis with a lower tendency to lymph node spread and better overall survival 87-89. Based on these characters, MMR has emerged as an important predictor of sensitivity for immunotherapy-based strategies 90, 91** (Figure 3).**

Moreover, MMR genes augment tumor immunity via affecting certain kinds of chemotherapies. Cancer chemotherapy was previously seemed as immune suppressive. Nowadays, chemotherapies are found to promote tumor immunity by disrupting strategies that tumors use to evade immune recognition 92. Different drugs can influence the immune response to cancer through a wide variety of mechanisms such as inducing immunogenic cell death, changing antigen-presentation, activating tumor cell targes and depleting immunosuppressive cells 93. It is known that MSI status of MMR gene may predict cancer response/resistance to certain chemotherapies. MMR deficient tumors are commonly resistant to methylating agents, platinum compounds and fluoropyrimidines 94, 95. A possible explanation is that under unrepairable DNA damage, DNA damage response proteins (i.e., ataxia telangiectasia mutated (ATM) and ataxia telangiectasia and Rad3-related protein (ATR)) are recruited by MMR proteins and induce cell cycle arrest, DNA repair, or apoptosis through DNA damage checkpoint proteins activation. While MMR deficiency might alter this mechanism and fail to remove these transferred cells. Accumulation of these DNA damage and transferred cells in turn confer resistance to many chemotherapies 96. Identifying the immunological changes associated with chemotherapy and MMR mutations is important in combining chemotherapy with checkpoint blockade and translating promising preclinical data into successful treatments for cancer patients.

MMR gene mutations are common in hematological malignancies and correlated with genome instability as well as tumorigenesis 97. Besides, chemotherapy-induced MMR mutations, such as thiopurine treatment, facilitate drug resistance in hematological malignant cells 98, 99. At present, immunotherapeutic methods targeting MMR are implicated in solid tumors. For example, two PD1-blocking antibodies, pembrolizumab and nivolumab, have shown efficacy in patients with metastatic colorectal cancers (CRCs) and have been granted accelerated FDA approval 100, 101. However, studies on hematologic malignancies are limited. Priyanjali et al. found 6 out of 536 plant derived biomolecules that may have anticancer properties against the tumors driven by deregulated MMR-pathways in blood-related cancers 102. Barthelemy et al. found that somatic deletions of 4 genes (FRAP1, HERC1, PRKCZ, PIK3C2B) recapitulated the MSH2 protein deficiency by enhancing MSH2-degradation, leading to significant reduction in MMR expression and increased resistance to thiopurines in human leukemia cells 41. Nuclear factor erythroid 2-related factor 2 (Nrf2, also called NFE2L2) overexpression protected acute myeloid leukemia (AML) cells from apoptosis induced by cytarabine via inhibiting MSH2 expression in a ROS-independent manner 103. Nikki et al. showed that MSH6 haploinsufficiency at relapse contributed to the development of thiopurine resistance in pediatric B-lymphoblastic leukemia 59. Other strategies such as upregulation of cGAS/STING, neoantigen-based vaccinations and immune checkpoint inhibitors could be effective ways to conquer MMR deficiency related tumors 39, 104.

## Prospect

There are at least six DNA repair pathways involved in repairing specific types of DNA damage: 1) the DNA MMR pathway repairs base-base mismatches and insertion/deletion mis-pairs; 2) the base excision repair (BER) pathway corrects single-strand breaks and homologous recombination (HR); 3) the non-homologous end joining (NHEJ) pathway repairs double-strand breaks; 4) the nucleotide excision repair (NER) pathway repairs DNA adducts; 5) the Fanconi anemia (FA) pathway fixes inter-strand crosslinks; 6) the O-6-methylguanine DNA methyltransferase (MGMT) pathway repairs O-6-methylguanine adducts 105. Among these pathways, mutational inactivation of MMR is the most typical and high-frequent character in tumor cells which allows cancer cells to accumulate thousands of mutations.

Nowadays, a plethora of drugs or drug combinations, including chemotherapy, targeted therapies, immunotherapies, immune checkpoint inhibitors, and CAR-T cells (chimeric antigen receptor-T cells) are applying to cancer treatment. However, malignant hematopoietic cells consistently develop cellular strategies to adapt to and survive from currently available therapies. This is regarded as a general hallmark and major drawback of leukemia and lymphoma, and significantly accounts for relapse and failure of treatments 106. Therefore, figuring out the mechanisms of resistance to therapies and finding new therapeutic targets are required in hematological malignancies treatment.

Epidemiological studies have shown that the occurrence of tumors is different among individuals, and the main determinant of this susceptibility is genetic differences 107. Somatic mutations in genomes happen when cells are exposed to mutational factors, including exogenous chemicals and physical agents as well as endogenous reactive metabolites such as reactive oxygen and nitrogen species (ROS and RNS) 108. Other sources of DNA damages are errors that occur during normal DNA metabolism or aberrant DNA processing reactions. Base substitutions, small insertions and deletions (indels), genome rearrangements and chromosome copy-number changes may happen during DNA replication, recombination, and repair 109. Thus, high-fidelity DNA replication is crucial to preserve the genomic integrity of eukaryotic cells and organismal health 110. Normal somatic cells possess DNA damage repair system to help them correct the mutations. While in most cancer cells, mutational inactivation of DNA repair genes was found which resulted in a profound repair defect and progressive accumulation of mutations throughout the genome 111.

The genomes of MMR deficient cancers are characterized by sequence alterations in microsatellites and thousands of mutations. These mutations are predicted to produce lots of mutation-associated neoantigens that might be recognized by the immune system and promote tumor destruction 112. Therefore, cancers with MMR deficiency were sensitive to PD-1 immune checkpoint blockade 113. Therapies with immune checkpoint inhibitors, such as anti-PD-1 antibodies, showed a potent and durable anti-tumor response regardless of the cancers' tissue of origin 114. Besides, the genetic diversity of MMR deficient cancers also influences the extension of anti-PD-1 immunotherapy response 115. In addition to immune checkpoint blockade, blocking the Nedd8-mediated degradation pathway with MLN4924 is another method to induce immunogenic cell death in MMR deficient cancer cells. Because of the proteome instability, an abundance of misfolded protein aggregates in MMR deficient tumors 116. To compensate, tumor cells utilize a Nedd8-mediated degradation pathway to facilitate clearance of misfolded proteins. Blocking this Nedd8 clearance pathway causes accumulation of misfolded protein, ultimately inducing immunogenic cell death 117. Abnormal activation of the cGAS-STING pathway due to the loss of MutLα-specific regulation of exonuclease 1 (Exo1) during DNA repair also facilizes the clearance of MMR deficient tumor cells 118.

Hematological malignancies are characterized by genetic defect in the form of chromosomal translocation or breakpoint/fusion, exposing cells to genomic instability 119. Loss of MMR function exacerbates genomic instabilities and results in the production of neoantigens 120. To escape form immunological surveillance, cancer cells highly express PD-1 antigen to suppress T cell function, which may contribute to refractory and relapsed acute myeloid leukemia 121. Therefore, applying checkpoint inhibitors is a promising strategy to combat cancer and improve survival rate through inducing genetic instability in cancer cells 119. Therapeutic cancer vaccines such as monoclonal proteasome-targeting antibodies, agonists for co-stimulatory molecules, adoptive transfer of genetically modified T cells like chimeric antigen receptor CAR-T cells 122, as well as agents that suppress negative regulatory pathways of T cells are all under active clinical investigation to provide longer survival time and fewer adverse reactions 123-125. Despite higher requirements and more challenges putting forward, we believe more new drugs will be coming out, bringing new treatment options to patients suffering from carcinoma all over the world.

## Figures and Tables

**Table 1:** Correlation between MMR genes and clinical characteristics in human cancer

Gene name	Gene Position	Tumor type	Related characteristics
MSH2	2p21-p16.3	Colorectal Cancer; NSCLC; Thyroid Cancer; Breast Cancer; Liver Cancer; et al.	Overall survival; Drug resistance
MSH3	5q14.1	Colorectal Cancer; **Prostate Cancer; Breast Cancer; Thyroid Cancer; Prostate Cancer;** Esophageal Cancer; Liver Cancer; et al.	Overall survival; Cancer risk; Chemotherapy resistance (Cisplatin, PARPi et al.)
MSH4	1p31.1	Head and neck Cancer; Thyroid Cancer; et al.	Anti PDL1 treatment; Overall survival
MSH5	6p21.33	Lung Cancer, non-Hodgkin's lymphoma	Cancer risk
MSH6	2p16.3	Colorectal Cancer; **Prostate Cancer; Breast Cancer; Thyroid Cancer; Prostate Cancer;** Esophageal Cancer; Pancreatic Cancer; Liver Cancer; et al.	Overall survival; Cancer risk; Chemotherapy resistance; Radiotherapy resistance; Immunotherapy;
MLH1	3p22.2	Colon Cancer; Gallbladder Cancer; Lung Cancer; Breast Cancer; Pancreatic Cancer; et al.	Metastasis; Overall survival; Chemotherapy resistance (TKI drug, PARPi et al.); Radiotherapy resistance; Immunotherapy
MLH3	14q24.3	Endometrial Cancer. Colorectal Cancer; Liver Cancer.	Overall survival; Cancer risk;
PMS2	7p22.1	Colorectal Cancer; **Prostate; Breast Cancer; Thyroid Cancer; prostate Cancer;** Esophageal Cancer; Pancreatic Cancer; Liver Cancer; et al.	Metastasis; Overall survival; Chemotherapy resistance; Radiotherapy resistance; Immunotherapy
PMS1	2q32.2	Colon Cancer; Breast Cancer; et al.	DNA repair; Cancer risk

## Abbreviations

MMR: mismatch repair
MSI: microsatellite instability
ROS: reactive oxygen species
RNS: reactive nitrogen species
BER: base excision repair
HR: homologous recombination
NHEJ: non-homologous end joining
NER: nucleotide excision repair
FA: Fanconi anemia
MGMT: O-6-methylguanine DNA methyltransferase
PD: programmed cell death
HNPPC: hereditary non-polyposis colorectal cancer
hMSH: Human mutS homolog protein
hMSL: Human mutL homolog protein
hPMS: Human PMS1 homolog protein
PMS: postmeiotic segregation increased
PCR: Polymerase chain reaction
SNP: single nucleotide polymorphism
RER: replication errors
LS: Lynch syndrome
AML: acute myeloid leukemia
Nrf2: nuclear factor erythroid 2-related factor 2
ATL: acute T-cell leukemia/lymphoma
GTBP: G/T mismatch binding protein
NF1: neurofibromatosis type 1
DLBCL: diffuse large B-cell lymphoma
APC: antigen-presenting cells
MAMPs: microbe-associated molecular patterns
DAMPs: damage-associated molecular patterns
DC: dendritic cell
MHC: major histocompatibility complex
CML: chronic myelogenous leukemia
ATM: ataxia telangiectasia mutated
ATR: ataxia telangiectasia and Rad3-related protein
CAR-T: chimeric antigen receptor-T cells
Exo1: exonuclease 1
